# Factors influencing plasticity in the arrival‐breeding interval in a migratory species reacting to climate change

**DOI:** 10.1002/ece3.5716

**Published:** 2019-10-16

**Authors:** Matthew Low, Debora Arlt, Jonas Knape, Tomas Pärt, Meit Öberg

**Affiliations:** ^1^ Department of Ecology Swedish University of Agricultural Sciences Uppsala Sweden; ^2^ WSP Sverige AB Uppsala Sweden

**Keywords:** arrival time, cumulative degree days, phenotypic plasticity, prebreeding period, prelaying interval, prelaying period, spring phenology

## Abstract

Climate change is profoundly affecting the phenology of many species. In migratory birds, there is evidence for advances in their arrival time at the breeding ground and their timing of breeding, yet empirical studies examining the interdependence between arrival and breeding time are lacking. Hence, evidence is scarce regarding how breeding time may be adjusted via the arrival‐breeding interval to help local populations adapt to local conditions or climate change. We used long‐term data from an intensively monitored population of the northern wheatear (*Oenanthe oenanthe*) to examine the factors related to the length of 734 separate arrival‐to‐breeding events from 549 individual females. From 1993 to 2017, the mean arrival and egg‐laying dates advanced by approximately the same amount (~5–6 days), with considerable between‐individual variation in the arrival‐breeding interval. The arrival‐breeding interval was shorter for: (a) individuals that arrived later in the season compared to early‐arriving birds, (b) for experienced females compared to first‐year breeders, (c) as spring progressed, and (d) in later years compared to earlier ones. The influence of these factors was much larger for birds arriving earlier in the season compared to later arriving birds, with most effects on variation in the arrival‐breeding interval being absent in late‐arriving birds. Thus, in this population it appears that the timing of breeding is not constrained by arrival for early‐ to midarriving birds, but instead is dependent on local conditions after arrival. For late‐arriving birds, however, the timing of breeding appears to be influenced by arrival constraints. Hence, impacts of climate change on arrival dates and local conditions are expected to vary for different parts of the population, with potential negative impacts associated with these factors likely to differ for early‐ versus late‐arriving birds.

## Graphical abstract

We examine the interrelationship between the timing of arrival and breeding in a migratory bird. The arrival‐breeding interval was related to the timing of arrival, the age of the bird, and the degree of spring progression and year. The timing of breeding appears to not be constrained by arrival date, but rather dependent on various local conditions.

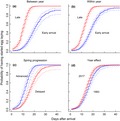

## 1 INTRODUCTION

For seasonally breeding species, the effect of climate change often shows as earlier breeding in association with warmer spring temperatures (Chambers et al., 2013; Parmesan, 2007). However, the degree of advancement in phenology may differ between trophic levels, leading to reduced breeding synchrony within some food webs. In birds, this may result in a phenological change between their timing of reproduction and the peak of food availability (Both, Bouwhuis, Lessells, & Visser, 2006; Both et al., 2010; Thackeray, Sparks, & Frederiksen, 2010), potentially affecting reproductive output and population growth rates (Both et al., 2006; Møller, Rubolini, & Lehikoinen, 2008; but see Reed, Jenouvrier, & Visser, 2013; Kristensen, Johansson, Ripa, & Jonzén, 2015). This issue is seen as especially important for migratory species that not only need to time their breeding to match lower‐trophic‐level phenology, but also their arrival at the breeding ground (Kristensen et al., 2015).

In migratory birds, a large number of studies have revealed that individuals possess notable flexibility both in arrival and breeding phenology in relation to warming spring temperatures (Charmantier & Gienapp, 2014; Møller et al., 2008; Parmesan, 2007; Saino et al., 2011), although in some cases arrival is less flexible and may constrain plasticity in breeding time (“arrival constraint hypothesis”; Both & Visser, 2001). Arrival and breeding phenology are often studied separately, but the period between arrival and breeding plays a crucial role in how populations track environmental change through breeding time adjustment (Crossin et al., 2010; Kristensen et al., 2015; Lany et al., 2016). If there is little flexibility in arrival times, individuals could still adjust the arrival‐breeding interval (a.k.a. prelaying period, being the time between arrival and the first egg being laid) to optimize their breeding time. Depending on the nature of the determinants of interval length, the arrival‐breeding interval could influence breeding phenology in a number of ways: For example, (a) if interval determinants are nonclimatic in origin (e.g., age, photoperiod, physiological ability), it may act as a constraint on breeding time advancement because these factors have a very limited range of phenotypic plasticity or may be tied to other independent factors; or (b) if influenced by environmental variables related to the local weather (e.g., temperature, food availability), it may enable adjustments of breeding time if birds alter their breeding to suit the prevailing conditions. There is evidence that the length of the arrival‐breeding interval can be influenced by a number of factors such as arrival time (shorter intervals with late arrival; Bêty, Gauthier, & Giroux, 2003; Potti, 1999; Teplitsky, Mouawad, Balbontin, De Lope, & Møller, 2011), age (although often correlated with arrival time; Potti, 1999; Wiebe & Gerstmar, 2010), individual condition (perhaps mainly for capital breeders; Bêty et al., 2003; Chastel, Weimerskirch, & Jouventin, 1995), photoperiod and physiology (Dawson, 2008), reproductive costs (Low, Arlt, Pärt, & Öberg, 2015), and local conditions (e.g., food availability and temperature; Högstedt, 1974; Wiebe & Gerstmar, 2010). However, it is not clear how these factors are interrelated, nor is it clear whether these factors allow birds to adjust their breeding time independent of arrival or in the context of climate change.

Many bird studies have shown advances in breeding time with increasing temperatures (Caro et al., 2013; Schaper et al., 2012; Visser, Holleman, & Caro, 2009; but see Nager & Noordwijk, 1992) or food availability (Jean‐Gagnon et al., 2018; Nager, Ruegger, & Noordwijk, 1997; Nilsson & Svensson, 1993). Yet, despite this demonstrated flexibility there are few empirical studies examining the interdependence between arrival and breeding time in migratory species (as modeled by Kristensen et al., 2015, but see Lany et al., 2016). Hence, evidence is still scarce regarding how breeding time is adjusted through changes in arrival time and/or the interval length between arrival and breeding. Thus, we still lack basic information on whether the main selecting forces on breeding time in migratory species are pre‐ or postarrival. According to a mathematical model by Kristensen et al. (2015), the optimal response in breeding time to changes in the environment depends on the relative timing and constraints of the processes affecting breeding time; these include arrival, seasonal resource (e.g., food) availability, and territory and resource acquisition. Studying the details of the breeding time processes is therefore crucial for understanding how migratory populations are able to track environmental change; however, empirical data are largely lacking.

**Figure 1:**
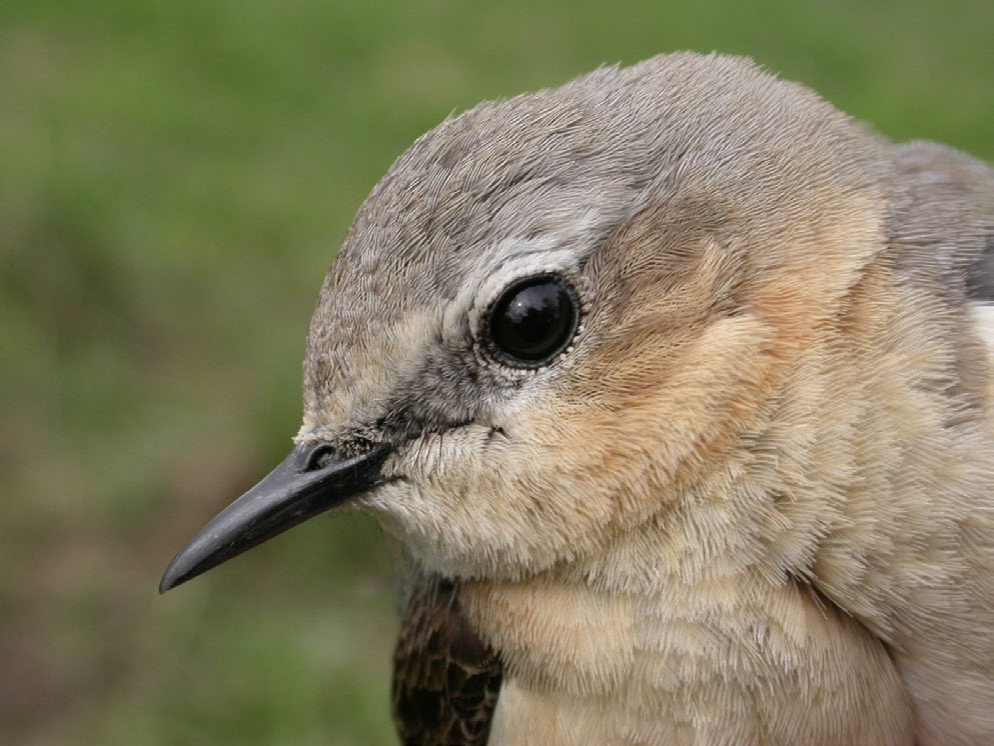
A female Northern Wheatear (*Oenanthe oenanthe*) at its Swedish breeding ground. Photo by Debora Arlt (used with permission)

To address this issue, we study a long‐distance migratory bird, the northern wheatear (*Oenanthe oenanthe*) that breeds in Sweden and overwinters in Africa (Figure 1). We investigate whether recent advances in its timing of breeding (Arlt & Pärt, 2017) were achieved by adjustment of arrival time, and/or the length of the arrival‐breeding interval. We also examine variation in the length of the arrival‐breeding interval using a Cox proportional hazards model to determine the factors related to the probability of initiating egg laying after arrival. We examine these factors not only to gain insights into recent phenological changes in this species, but also to gain a general understanding of the factors that influence or constrain the timing of breeding in migratory birds. Thus, we ask the following questions: (a) How are recent changes in the patterns of breeding time in the northern wheatear (Arlt & Pärt, 2017) reflected in changes in its arrival times and the arrival‐breeding interval; (b) What factors are related to the probability of beginning egg laying after arrival (e.g., we expect female age, the timing of arrival and the degree of spring progression to influence the arrival‐breeding interval); and (c) How does the relative importance of these factors change throughout the breeding season? Like many seasonally breeding organisms the wheatear displays a seasonal decline in fitness related to breeding time (Öberg, Pärt, Arlt, Laugen, & Low, 2014; Pärt, Knape, Low, Öberg, & Arlt, 2017); thus, we would expect factors determining the length of the arrival‐breeding interval to vary in their importance depending on the timing of arrival.

## 2 METHODS

### 2.1 The wheatear study system

The northern wheatear (hereafter “wheatear”) is an insectivorous, cavity‐nesting passerine that in our study area breeds in open farmland habitats. Our study area is ~60‐km^2^ heterogeneous agricultural landscape situated southeast of Uppsala in southern central Sweden (59°50′N, 17°50′E) and consists of ~230 territory sites that have been occupied by wheatears at least once since 1993; each year 120–180 pairs breed in the area. In a smaller core area (~40 km^2^, 150 sites, 80–90 pairs per year), each territory site has been visited at least every 2–5 days throughout the breeding season to collect detailed data on demographic parameters. Every year we ringed nestlings and adults with a unique combination of color rings and a numbered aluminum ring (adults from ~60% of all breeding attempts, nestlings from ~90% of all successful breeding attempts). This allowed us to monitor the relationship between arrival and breeding for hundreds of individuals.

Wheatears arrive at the study area during April, with males tending to arrive 3 days earlier than females (Arlt & Pärt, 2008), and older breeders 6 days earlier than 1‐year‐old breeders of the same sex (D. Arlt, unpublished data). Individuals are usually discovered at breeding territory establishment where birds appear to settle at the day of their arrival (we rarely observed transient birds moving between sites). We defined arrival date as the date when an individual was first observed in the study area (relative to 1st May). Those observed arrival dates closely corresponded to arrival dates estimated from geolocation data from a smaller sample of birds (*N* = 12, median difference of 2 days [range 0–4]; Arlt, Olsson, Fox, Low, & Pärt, 2015). We collected arrival and breeding data during 20 years (1993–1996 and 2002–2017 [arrival data were not routinely collected from 1997–2001]) and only included data from areas where territories were frequently visited (every 2–3 days) during the arrival period, from early April to 10th May. We used female data because egg laying and incubation are female‐specific traits and timing of breeding therefore should be largely determined by the female (Caro, Charmantier, & Lambrechts, 2009). This provided us with 734 separate arrival‐to‐breeding events from 549 individual females where we had accurate data on their arrival and timing of breeding.

The earliest individuals began egg laying in early May. While some nests were found during egg laying/incubation, the majority (~80%) were found after hatching and, therefore, the timing of breeding was estimated based on chick age (comparing morphological development to photos of known hatch‐date nestlings of different ages), brood/clutch size (assuming egg laying of one egg per day on consecutive days), and 13 ± 1 days of incubation, with incubation beginning when the penultimate egg was laid (Öberg et al., 2014). We only include data from first (initial) breeding attempts and calculated the arrival‐breeding time interval as the number of days between arrival and the estimated start of egg laying. Most birds in the study area attempt breeding only once per season (~93%); however, it is possible that some birds could have eluded observation during a failed first attempt and their recorded breeding date is from a second attempt. It is also possible that some very late‐arriving birds were considered as “unknown” or as “second attempts” and incorrectly excluded from the data. While this would bias the data in those specific cases (e.g., into having a later arrival or longer prelaying interval than actually occurred), these cases unlikely total >1% of all observations considering the search effort and the clear behavioral signs of breeding. Thus, we consider our results robust to these sources of observation error. Based on plumage characteristics, we distinguished two age categories in breeding adults, 1 year old (first‐year breeder) or older (Pärt, 2001).

### 2.2 Weather data

We obtained data on daily average temperature (°C) from the Ultuna Climate Station (59°82′N, 17°65′E; http://grodden.evp.slu.se/slu_klimat/index.html) ~10 km from the center of our study area. Plant and arthropod development early in the season is strongly determined by degree days, that is, temperature exceeding a threshold (base) temperature below which growth and development rate is zero. Insect phenology models are usually based on accumulated degree‐days (i.e., the cumulative sum of degree days during a time period; Hodgson et al., 2011; Nietschke, Magarey, Borchert, Calvin, & Jones, 2007). We therefore used thermal sum as an indicator of spring phenology: with degree days = *daily mean temperature − base temperature*, and thermal sum for each day calculated as the cumulative sum of degree days since January 1 in each year. We used a base temperature of 5°C, which is the threshold used by the Swedish Meteorological and Hydrological Institute (https://www.smhi.se) to analyze the start of the growing season in agricultural areas (Persson, Bärring, Kjellström, Strandberg, & Rummukainen, 2007).

### 2.3 Statistical analysis

#### 2.3.1 Temporal trends in arrival, breeding, and the arrival‐breeding interval

We investigated how the timing of arrival, breeding, and the duration of the arrival‐breeding interval have changed during the study period. All regression models used a normal response distribution and included: yearly trend (continuous) and female age (first‐year breeder or experienced) as explanatory variables, with female identity included as a random intercept to account for individuals who were sampled in >1 year (Appendix S1a). For changes in the arrival‐breeding interval, we modeled this in terms of the year effect during the study period (as above), as well as a second model that also accounted for the arrival date (interval ~ intercept [ID] + year + age + arrival + arrival × year). These two ways of modeling the interval provided us with subtly different interpretations for its change over time. The first gives information as to the absolute magnitude of change in the interval and reflects relative changes in arrival versus breeding date across the years of the study. Thus, if the interval is declining over time, this suggests something may be constraining arrival relative to the timing of breeding; if the interval is increasing, then breeding may be constrained relative to arrival. However, this only looks at the relationship between arrival and breeding, and it does not account for shifts in the overall timing of the breeding season. The second modeling approach examines the change in the interval for birds when the arrival date has been accounted for. A declining interval over time here would show whether birds have been able to reduce their interval relative to the absolute date and the factors linked to that, rather than simply relative to arrival. Models were implemented in a Bayesian framework (JAGS; Plummer, 2015), with the probability and magnitude of change directly calculated from the posterior distributions of derived variables (Appendix S1a,b). We also modeled the variance in egg‐laying dates relative to arrival to examine whether early‐arriving birds have more flexibility in their arrival‐breeding interval compared to late‐arriving birds (e.g., Teplitsky et al., 2011); here, we included a regression model on sigma [sigma ~ intercept + arrival + arrival^2^] in addition to the model on the mean [mean ~ intercept + age + year] (Appendix S1c).

To check whether any trends we observed were the result of sampling biases within our study population (since we only have arrival‐breeding data for a fraction of our study birds, and we have more arrival data in some years than others), we compared changes in the timing of breeding from our sample (with matched arrival dates) to changes in the timing of breeding for all known‐aged birds in our study area that we had breeding data for (*n* = 2,368). The yearly trend from a regression model that accounted for female age and female identity did not differ between the two groups (“year” regression coefficient −0.205 ± 0.03; *n* = 734 vs. −0.208 ± 0.03; *n* = 2,368), strongly suggesting our arrival‐breeding data did not suffer from sampling bias in this regard.

#### 2.3.2 The arrival‐breeding interval

**Figure 2:**
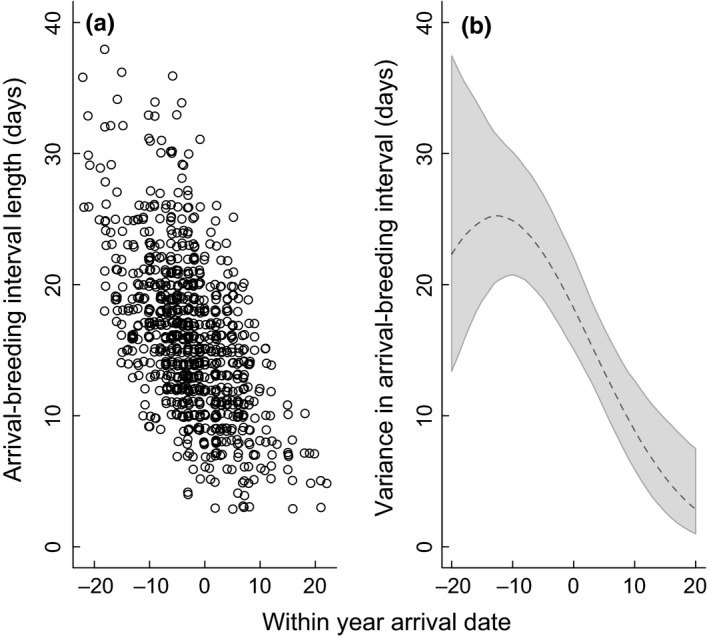
The relationship between within‐year arrival relative to the year‐specific mean arrival date (= 0 on *x*‐axis) and: (a) the length of the arrival‐breeding interval (days after arrival), and (b) the variance in the arrival‐breeding interval (days; mean [dashed line] and 95% CIs [gray shading]). Each point in the left panel represents an individual arrival‐breeding event and is “jittered” so that all raw data points can be seen in the plot. The gray shading in the right panel shows the 95% credible intervals of the predicted variance (days) in the timing of the first egg being laid relative to the arrival date (i.e., the arrival‐breeding interval) for the same individual data shown with the points in the left panel (Appendix S1c for a full model description)

To better understand the determinants of the arrival‐breeding interval, we also analyzed it using a more detailed survival model. Survival or time‐to‐event analyses are a suite of statistical methods used to analyze the occurrence and timing of events. The Cox proportional hazards regression model (hereafter “the Cox model”; Cox, 1972) is a survival model that describes the instantaneous rate at which subjects experience events, given that the event has not yet happened to the subject. In this case, we wanted to model the probability that an individual bird would begin egg laying in the days following arrival. Thus, this modeling framework allowed us to explore the factors related to variation in the arrival‐breeding interval (this variation is clearly evident in Figure 2a). Because covariates in the Cox model may be fixed within breeding events or vary with time‐to‐event (i.e., daily), we could examine factors that varied not only between years and individuals, but also varied on a daily basis.

We analyzed the probability of starting egg laying on any given day after arrival (i.e., the arrival‐breeding interval) for 734 breeding attempts from a total of 549 females for which we knew arrival date, the timing of egg laying and had all covariate information we were interested in. This provided us with a total of 12,078 interval‐days, where we could model the instantaneous probability that a given female would lay her first egg at time *t* given that she had not already begun egg laying. Because females were repeated within the dataset, we used an extension to the Cox model that included female ID as a random effect (a so‐called “frailty model”; Therneau & Grambsch, 2000). Without this random effect, the Schoenfeld's residuals indicated some violation of the proportional hazards assumption. We examined the following covariates: (a) the individual arrival date centered relative to that year's mean arrival, (b) the mean arrival date for each year to account for year effects influencing arrival and subsequent impacts on the arrival‐breeding interval, (c) the progression of spring (daily thermal sum) for each date during the study period, (d) year as a linear change during the study period, as a measure of progressive climate change independent of other measures included in the model, and (e) the age of the female (first‐year breeder or older). We also modeled all two‐way interactions that included the individual arrival date to examine how the relative influence of the different covariates changed relative to the timing of arrival in each year. This was because the arrival‐breeding interval showed a dramatic reduction in variation with later arrival dates (Figure 2b), suggesting strong covariate interactions were associated with this variable. In our study area, there are strong associations between habitat and demography (Arlt, Forslund, Jeppsson, & Pärt, 2008; Low, Arlt, Eggers, & Pärt, 2010; Parquet et al., 2019); however, these were unlikely to influence the arrival‐breeding interval because they are associated with changes in field‐layer height later in the season (i.e., during chick rearing). Thus, we did not include a territory quality variable in our analyses; subsequent confirmatory analyses supported this decision and showed that territory field‐layer height had no relationship with the arrival‐breeding interval (all *p*‐values >0.5). Analyses were done in R (R Core Team, 2019) using the “coxme” and “simPH” packages (Gandrud, 2015; Therneau, 2018).

## 3 RESULTS

### 3.1 Arrival and breeding time

**Figure 3:**
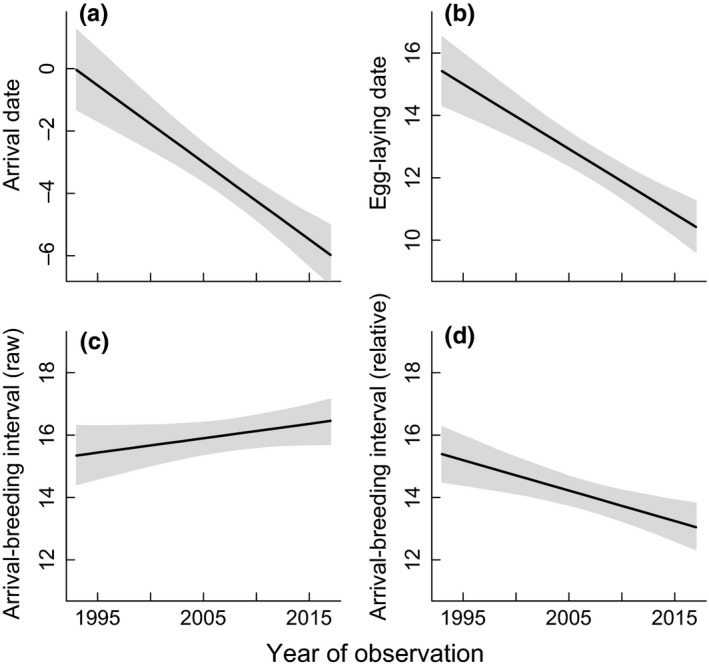
Estimated changes in phenology for northern wheatears from 1993 to 2017 for: (a) arrival (relative to 1st May = 0), (b) the initiation of egg laying (also relative to 1st May), (c) the raw arrival‐breeding interval (days from arrival to initiation of egg laying), and (d) the arrival‐breeding interval when arrival date had been accounted for in the regression model (days). Predictions are derived from Bayesian regression models and show the mean (black line) and 95% credible intervals for the mean (gray shading)

Females arrived during ~45 days between early April and mid‐May during the years of the study. Arrival dates were strongly positively correlated with egg‐laying dates and negatively correlated with variance in breeding dates and the length of the arrival‐breeding interval. Early‐arriving birds had much longer arrival‐breeding intervals than late‐arriving birds (mean of ~25 vs. 5 days, respectively, with the interval becoming 0.5 days shorter for each 1‐day delay in arrival; coefficients from GLMM: intercept = 14.8 ± 0.49, arrival date slope = −0.51 ± 0.02 days) and demonstrated much more flexibility in their arrival‐breeding intervals (variance of ~25 vs. 4 days, respectively; Figure 2). Between 1993 and 2017 arrival dates advanced slightly more than egg‐laying dates (6 vs. 5 days), meaning that the raw arrival‐breeding interval increased by ~1 day over this period (Figure 3; Table 1). However, if changes in the arrival dates were also included in the model, then the yearly trend in the arrival‐breeding interval showed a decrease in 2.3 days from 1993 to 2017 (Figure 3; Table 1). This indicates that a bird arriving on the same calendar date in early years of the study had a longer arrival‐breeding interval compared to one arriving in later years.

**Table 1:** The mean difference (in days) for arrival, egg‐laying, and the arrival‐breeding interval from the start of the study (1993) to the end of the study (2017)

Factor	Mean ± *SD*	95% CIs	*p* (2017 < 1993)
Arrival date	−6.14 ± 0.91	−4.2 to −7.8	1
Egg‐laying date	−4.92 ± 0.78	−3.4 to −6.4	1
Arrival‐breeding interval (raw)	0.99 ± 0.73	−0.5 to 2.3	.059
Arrival‐breeding interval (arrival adjusted)	−2.33 ± 0.69	−1.0 to −3.7	.995

Note

The estimates are the mean ± *SD* and 95% credible intervals of the posterior distribution of the difference of 2017 relative to 1993. Thus negative estimates show that arrival and egg‐laying dates were earlier in 2017, and the raw arrival‐breeding interval was longer than in 1993. The *p* (2017 < 1993) shows the probability that values in 2017 were earlier (or shorter) than in 1993.

### 3.2 Factors explaining the length of the arrival‐breeding interval

**Figure 4:**
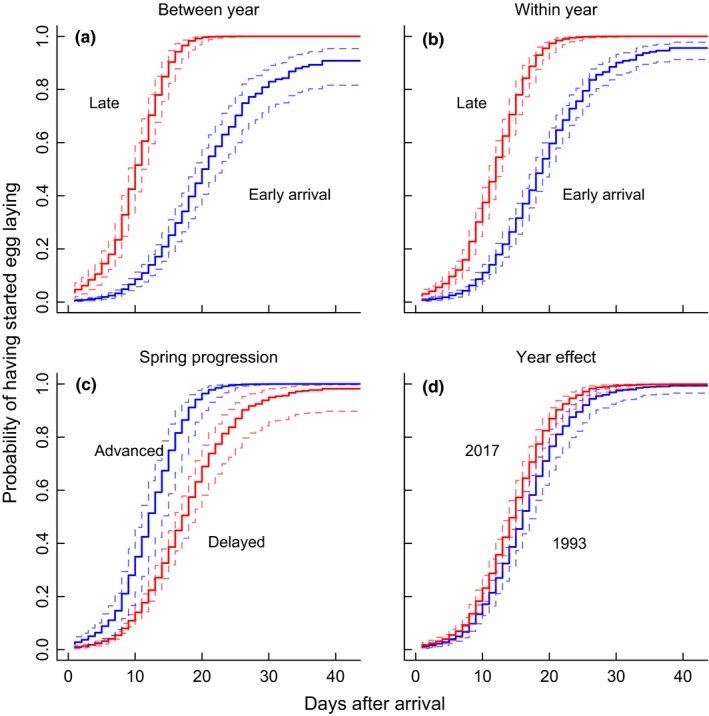
The relationship between the time since arrival at the breeding ground and the probability of initiating egg laying for: (a) birds arriving in “early” arrival years versus “late” arrival years, (b) individuals arriving early versus late relative to the mean within‐year arrival date, (c) the degree of spring progression (daily thermal sums) for each day after arrival, and (d) the year effect from 1993 to 2017. Plots are predicted means with their associated 95% CIs from the full Cox proportional hazards model, and each prediction shows the range of variation within that factor when all other variables are held at their mean values

**Figure 5:**
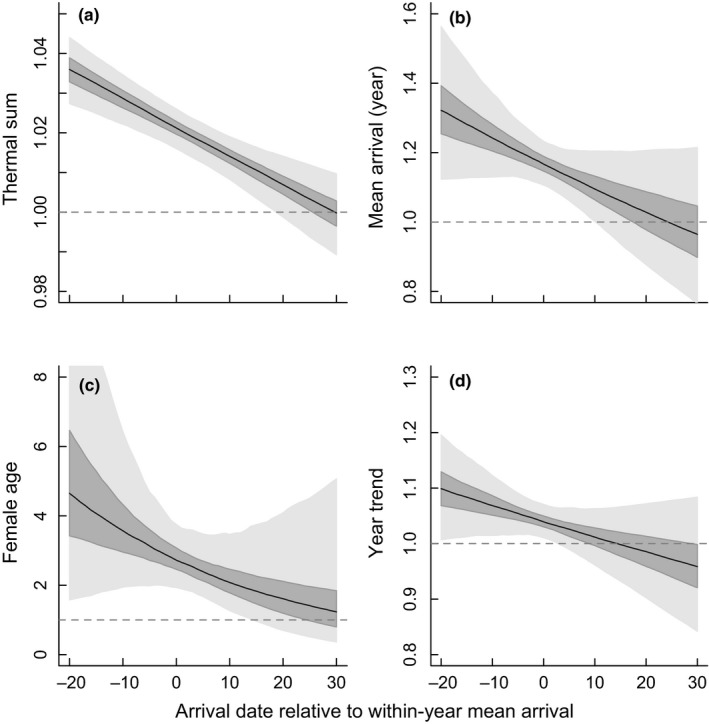
The interaction effects (i.e., changes in the marginal effect on the hazard from the full Cox model) of the different factors relative to the within‐year arrival date (a = spring progression/thermal sum; b = between‐year mean arrival effects; c = female age [experienced female effect], and d = year trend from 1993 to 2017) when all other factors are held at their mean values. The *y*‐axes show the exponentiated coefficient for that factor for different arrival dates, with all of these effects becoming less influential for late‐arriving birds. The horizontal dashed line at 1.0 is the point where the factor has no effect on the probability of egg laying (because the exponentiated coefficient has a multiplicative effect on the hazard). The black line shows the median, the dark shading the 50% CIs, and the light shading the 95% CIs from 1,000 bootstrap simulations per arrival date (using the “simPH” package in R)

The probability of initiating egg laying was related to arrival time at the breeding grounds (from both the between‐ and within‐year perspective), female age, degree of spring progression, and year effect (Figure 4; Table 2). Thus, the arrival‐breeding interval was shorter: (a) for birds that arrived in “late” years compared to “early” years, (b) for individuals that arrived later in the season compared to early‐arriving birds, (c) for experienced females compared to first‐year breeders, (d) as spring progressed, and (e) in 2017 compared to 1993 (Figure 4). The influence of these effects on the probability of initiating egg laying was much larger for early‐arriving birds compared to late‐arriving birds (Figure 5; Table 2), with most effects on variation in egg‐laying probability being absent in late‐arriving birds (Figure 5) probably because variation in the arrival‐breeding interval was largely absent at this time (Figure 2).

**Table 2:** Regression coefficients (Coeff) ± Standard Errors (*SE*) and the exponentiated coefficients (exp (Coeff)) and their 95% CI range from the full Cox proportional hazards model

Factor	Coeff ± *SE*	Exp (Coeff)	95% CI range	*p*
Arrival_BETWEEN YEAR_	0.157 ± 0.027	1.17	1.10–1.23	<.001
Arrival_WITHIN YEAR_	0.280 ± 0.048	1.32	1.20–1.45	<.001
Female age	1.012 ± 0.173	2.75	1.95–3.86	<.001
Spring progression	0.021 ± 0.002	1.02	1.01–1.02	<.001
Year effect	0.038 ± 0.014	1.04	1.01–1.07	.006
Arr_WITHIN_ × Arr_BETWEEN_	−0.006 ± 0.003	0.993	0.986–1.001	.10
Arr_WITHIN_ × Age	−0.026 ± 0.022	0.974	0.932–1.018	.25
Arr_WITHIN_ × Spring	−0.0007 ± 0.0001	0.999	0.999–0.999	<.001
Arr_WITHIN_ × Year	−0.003 ± 0.002	0.997	0.993–1.001	.19

Note

An exponentiated coefficient larger than 1 is interpreted such that there is a positive multiplicative effect of the covariate on the hazard (i.e., the probability of initiating egg laying increases with that factor, thus yielding a shorter interval between arrival and egg laying). For categorical covariates (i.e., female age), the exponentiated coefficient shows the effect of breeder experience relative to the hazard in the reference group (inexperienced females). For the other covariates, it shows the increase/decrease in the hazard for a one‐unit increase in the covariate. *p*‐values are also shown.

## 4 DISCUSSION

Predicting whether migratory birds will alter their arrival and egg‐laying dates in the context of climate change is challenging, because they face a suite of trade‐offs relating to early‐ and late‐season processes such as: adult and offspring survival, competition for breeding sites, and resource acquisition for egg‐laying and subsequent offspring feeding (Johansson & Jonzén, 2012; Kristensen et al., 2015). Thus, the historical phenology of a population and its phenological responses to climate change will depend on the relative timing and change of these processes (Kristensen et al., 2015). Previous findings from this population questioned whether earlier breeding but declining demographic rates in this population result from “mismatched” phenologies or a general deterioration in environmental quality (Arlt & Pärt, 2017). However, without individual arrival data this picture is incomplete, as it is unclear whether some (or all) of the change in the timing of breeding relative to spring progression is related to constraints associated with arrival (e.g., Kristensen et al., 2015). Here, we investigated both arrival time and breeding time and their interrelation. This showed that yearly trends of breeding time, and the factors related to variation in the arrival‐breeding interval, were largely independent of arrival time for early‐ to midarriving birds in our study population. Instead, and in line with Arlt and Pärt (2017), adjustments of breeding time appear most likely constrained by seasonally limited availability of resources.

### 4.1 Yearly trends in arrival and breeding

Both arrival and egg‐laying dates advanced by almost a week during the past 25 years with no indication of a temporal trend for females reducing their arrival‐breeding interval during the study, suggesting that these two measures of phenology were interrelated. The idea is that in order for wheatear females to breed earlier and track earlier spring progression (i.e., the seasonal “food peak” for their offspring), they need to arrive earlier because they cannot make the necessary breeding time adjustments by simply shortening their arrival‐breeding interval. However, if we examine the arrival‐breeding interval data relative to the within‐year arrival date (Figure 2), we see that early‐ to midarriving birds appear to have a lot of potential to reduce their arrival‐breeding interval. Early arrivers took on average almost 4 weeks to start egg laying compared to late arrivers that started egg laying within a week. Early‐to‐mid arrivers also had a much larger variation in their interval compared to late arrivers. Since late‐arriving birds demonstrate that wheatears have the ability to begin laying soon after arrival, it begs the question of why most birds simply did not adjust their timing of breeding independent of their arrival time (e.g., cases 1 and 2 in Kristensen et al., 2015)? We explore three possibilities for this.

The first is that the cues the birds use along their migration route to determine their arrival time were largely in synchrony with seasonal changes for initiating egg laying at the breeding ground. Thus, birds arrived earlier during the course of the study and there was no requirement for birds to further adjust their breeding time, and hence arrival‐breeding interval, beyond what was normal for this population (i.e., the arrival adjustment “pulled” the breeding time along with it). One potential problem with this explanation is that the arrival and breeding dates have not been keeping up with advances in spring progression at the breeding ground (Arlt & Pärt, 2017), meaning that birds are arriving progressively later relative to the advancement of spring, but then taking almost the same amount of time before they decide to breed. Assuming that spring progression influences the timing of initiating breeding, this suggests there are other factors influencing breeding decisions.

Second, although the arrival‐breeding interval shows a large amount of within‐year and within‐individual variation suggesting that birds could adjust breeding independent of arrival date, this variation may be linked to the availability of seasonally limited essential resources (e.g., calcium; Reynolds, Mänd, & Tilgar, 2004; but see Wilkin, Gosler, Garant, Reynolds, & Sheldon, 2009), energy (Hennin et al., 2016; Visser & Hollemann, 2001), and/or the condition of the female at arrival (Jean‐Gagnon et al., 2018; Rowe, Ludwig, & Schluter, 1994; Verhulst & Nilsson, 2008). Thus, the limited availability of essential nutrients early in the season prevents females from earlier breeding because of its fitness impacts on their reproductive output or survival. Here, early‐arriving birds and young birds (less experienced or in poorer condition) have a longer arrival‐breeding interval because they need a longer time to accumulate these nutrients before breeding, while late‐arriving birds have these nutrients in abundant supply and can breed almost immediately. This would explain the patterns in Figure 2 where early arrivers took much longer to breed, presumably because of low resource availability. Also, birds early in the season had much greater variation in their arrival‐breeding interval, either because females arrived in a range of conditions during that time, or the impact of female condition on the arrival‐breeding interval was only a constraint early in the season.

One factor likely linked to female breeding condition is the state of their reproductive system at arrival. The avian uterus largely regresses during nonbreeding for flight efficiency reasons and needs to regrow prior to breeding (Dawson, King, Bentley, & Ball, 2001), which may take substantial time and resources (Gwinner, 1987; Williams, 2012). Although overlaps between migration and development of the reproductive tract is a relatively unexplored area, there is some evidence that reproductive regrowth begins during migration (Ramenofsky & Wingfield, 2006) and that physiological breeding changes occur during migration for males (Covino, Jawor, Morris, & Moore, 2018). Because migration is energetically demanding and birds will pay a cost for carrying unnecessary weight (Alerstam, 2011; Kullberg, Fransson, & Jakobsson, 1996), the pressure for early arrival is likely to invoke a trade‐off between early arrival and gonadal development (Crossin et al., 2010; Williams, 2012). Thus, early‐arriving females may be less reproductively ready at arrival (Williams, 2012) as they could be expected to encounter less favorable conditions during their migratory journey, potentially limiting their resource availability during migration and this preventing them from simultaneously arriving early and rapidly advancing the development of their reproductive tract. In contrast, late‐arriving individuals migrate during more favorable conditions, which could allow them to reach the breeding area with a highly developed breeding capacity and would explain their ability to rapidly initiate breeding after arrival with little between‐individual variation (but see Covino et al., 2018 for data showing that female hypothalamic–pituitary–gonadal sensitivity was not higher for late migrating birds).

A third explanation for why the birds in our study appeared to adjust their arrival and breeding dates rather than the arrival‐breeding interval is that arrival time and breeding time were not interrelated at all, but result from two simultaneous processes. The first process is driving earlier arrival to secure the best territories (as there is a strong link between territory quality and demography in this system; Arlt et al., 2008; Arlt & Pärt, 2007; Low et al., 2010; Öberg et al., 2014; Parquet et al., 2019). Here, earlier springs may have relaxed the survival cost of early arrival, given that arrival date is primarily a compromise between early‐season survival and nest‐site competition (Kristensen et al., 2015). The second process involves an adjustment of the arrival‐breeding interval as a result of optimizing breeding time to the local conditions (see also Lany et al., 2016). Thus, rather than seeing breeding time being pulled forwards or backward by some constraints imposed by arrival date (e.g., Figure 3c), here we consider the arrival‐breeding interval being adjusted to compensate for the earlier arrival dates that are being driven by nest‐site competition (Figure 3d). In this explanation, breeding is not constrained by arrival at all, and the only reason we do not see trends in the arrival‐breeding interval is that these trends are masked by simultaneous trends in the arrival date (Figure 3a–d).

### 4.2 Factors related to the arrival‐breeding interval

The time‐to‐event analyses reinforced the importance of arrival date as a key determinant of the arrival‐breeding interval. Between‐ and within‐year early arrivers had consistently lower probabilities of initiating egg laying compared to late arrivers (Figure 4), with the probability of egg laying being much higher for late arrivers for any given day after arrival. There was also an additional effect of spring progression and female age, with females in years with earlier springs and older females having a higher probability of initiating egg laying. Experienced females had shorter arrival‐breeding intervals compared to young females (see also Potti, 1999; Teplitsky et al., 2011), indicating that interval length may be affected by differences in individual or habitat quality (as experienced females arrive earlier [Arlt & Pärt, 2008] and will choose preferred territories). Although late‐arriving birds may have their breeding dates constrained by their time of arrival, this arrival constraint appears unlikely for early‐arriving birds. Early and midarrivers appear to wait until spring conditions have progressed to a certain stage, or seasonally limited resources have become available in sufficient quantity before initiating breeding (cf. Hennin et al., 2016; Lany et al., 2016). This further supports the conclusions in Arlt and Pärt (2017) that declining trends in demography in this population result from a general decline in habitat quality over the study area, rather than the timing of breeding becoming “mismatched” to local seasonal conditions. This is one reason why the arrival‐breeding interval appeared to be increasing rather than decreasing over time; it may be that in later years in has become more difficult to find the resources necessary to breed.

Interestingly, the importance of these effects on the probability of egg laying was conditional on the timing of within‐season arrival (Figure 5). Variation in year effects (including between‐year mean arrival), spring progression and female age all influenced the arrival‐breeding interval of early arrivers, with this effect declining during the season and having no effect on late arrivers. The drivers behind this pattern are likely the relative changes in resource availability, which relate to the fitness impacts of delaying breeding (Drent & Daan, 1980). Here, early‐arriving birds will face relatively poor local conditions and so will be more influenced in their decision to breed by their own condition, local territory quality, and the uncertainty about fitness trade‐offs of initiating versus delaying breeding. Compare this with a late‐arriving bird, where conditions are generally good because spring has progressed to beyond a certain threshold and there is much more certainty that breeding needs to be initiated sooner rather than later. Thus, they all begin breeding soon after arrival, with very little between‐individual variation (Figure 2). These patterns suggest that breeding time for the majority of the population is not constrained by arrival date, and that the arrival‐breeding interval still provides the flexibility they need to time their breeding to suit local conditions.

We show that both arrival and breeding time are somewhat flexible and that breeding time is generally not constrained by arrival, at least for early‐ to midarriving birds. Still, adjustments of breeding time seem constrained by local seasonally limited availability of essential resources (i.e., there are both pre‐ and postarrival factors acting on selection for the timing of breeding in this population). Although arrival and breeding in this population now occur at a relatively later stage of spring progression because of climate change, it is not clear that this has resulted in some sort of “mismatch” between breeding and the peak food supply, nor that the birds are somehow constrained in their breeding time because they are arriving at the breeding ground too late. It seems likely for early‐ to midarriving birds that their timing of arrival and breeding are largely independent, and instead depend on local conditions after arrival to determine their breeding time. There is an advantage for birds to arrive earlier now compared to 20 years ago because of the relative advantages of securing the best territories (i.e., nest‐site competition) in combination with reduced costs of arriving early as spring temperatures have warmed. For late‐arriving birds, however, the timing of breeding appears to be influenced by arrival constraints. Hence, impacts of climate change on arrival dates and local conditions are expected to vary for different parts of the population, with potential negative impacts associated with these factors likely to differ for early‐ versus late‐arriving birds. Thus, population responses of migratory birds to climate change may be unpredictable and arise from multiple factors differentially operating on subsections of the population.

## CONFLICT OF INTEREST

None declared.

## AUTHORS CONTRIBUTIONS

MÖ, ML, TP, DA, and JK involved in the study conception and design. DA, MÖ, and TP performed the data collection. ML, MÖ, and JK involved in analysis. ML led the writing of the manuscript with MÖ and DA. All authors contributed critically to the drafts and gave final approval for submission.

## Data Availability

Data are available via the Dryad Digital Repository DOI: https://doi.org/10.5061/dryad.2g6v864
