# Supplementary material for: Factors influencing plasticity in the arrival‐breeding interval in a migratory species reacting to climate change
Source: Ecol Evol. 2019 Oct 16;9(21):12291–301. doi: 10.1002/ece3.5716 (PMC6854385; doi:10.1002/ece3.5716)
Supplement: Supplementary file 1 [file ECE3-9-12291-s001.pdf]

Supplementary Appendix S1 for the paper:

Factors influencing plasticity in the arrival-breeding interval in a migratory species reacting to climate change

by Matthew Low, Debora Arlt, Jonas Knape, Tomas Pärt & Meit Öberg

This document contains the formal model specifications and the JAGS code for the Bayesian models implemented in this paper. Specifically formal model specifications and the JAGS model code for the models relating to the following analyses:

Appendix 1a: for Figs 2a, 2b & 2c in main MS for main regression models

Appendix 1b: for Fig. 2d in main MS

Appendix 1c: for Fig. 1 in main MS based on variance modelling

For all Bayesian models model fit was checked using post-predictive checks by simulating the data back from the model and comparing it to the data used to construct the model. In all cases the mean and the variance indicated reasonable model fit (Bayes P between 0.1 and 0.9).

Appendix 1a

1. Formal model specification for the year trend in arrival, egg laying date and the raw arrival-breeding interval (Fig. 2a, b, c). This is a regression where date is related to female age and a yearly trend, while accounting for repeated female observations via a random intercept (i = individual observations, j = female id).

$$date_i \sim Normal(\mu_i, \sigma)$$

$$\mu_i = \alpha_j + \beta_1 age_i + \beta_2 year_i$$

$$\alpha_j \sim Normal(\mu_\alpha, \sigma_\alpha)$$

2. JAGS code for the above model

```
model{
for(i in 1:n.obs){

date[i]~dnorm(mu[i],tau)
mu[i]<-a[id[i]] + b1*age[i] + b2*yr[i]
}

#random intercept
for(j in 1:nobs.id){
a[j]~dnorm(mu.id, tau.id)
}

#priors
mu.id~dnorm(0, 0.001)
b1~dnorm(0,0.001)
b2~dnorm(0,0.001)
tau.id<-1/sigma.id^2
tau<-1/sigma^2
sigma.id~dunif(0,20)
sigma~dunif(0,20)
}#close
```

Appendix 1b

1. Model for the arrival adjusted arrival-breeding interval (Fig. 2d). This regression is where date is related to female age, a yearly trend and arrival date, while accounting for repeated female observations via a random intercept (i = individual observations, j = female id).

$$\text{interval}_i \sim Normal(\mu_i, \sigma)$$

$$\mu_i = \alpha_j + \beta_1 age_i + \beta_2 year_i + \beta_3 arrival_i + \beta_4 arrival_i * year_i$$

$$\alpha_j \sim Normal(\mu_\alpha, \sigma_\alpha)$$

2. JAGS code for the above model

```
model{

#likelihood

for(i in 1:n.obs){
int[i]~dnorm(mu.int[i],tau.int)
mu.int[i]<-a.int[id.int[i]] + b1.int*age[i] + b2.int*yr[i] +
 b3.int*arr[i] + b4.int*yr[i]*arr[i]
}

#random intercept
for(j in 1:nobs.id){
a.int[j]~dnorm(mu.id.int, tau.id.int)
}

#priors
mu.id.int~dnorm(0, 0.001)
b1.int~dnorm(0,0.001)
b2.int~dnorm(0,0.001)
b3.int~dnorm(0,0.001)
b4.int~dnorm(0,0.001)

tau.id.int<-1/sigma.id.int^2
tau.int<-1/sigma.int^2
sigma.id.int~dunif(0,20)
sigma.int~dunif(0,20)

 }#close model
```

Appendix 1c

1. Model for the relationship between arrival date and variance in the arrival-breeding interval (see Fig. 1). This is a regression where date is related to female age and a yearly trend, while accounting for repeated female observations via a random intercept (i = individual observations, j = female id). This model has an additional equation on sigma, to allow sigma to vary according to the arrival date.

$$\text{interval length}_i \sim Normal(\mu_i, \sigma_i)$$

$$\mu_i = \alpha_j + \beta_1 age_i + \beta_2 year_i$$

$$\sigma_i = \alpha_{sigma} + \beta_3 arrival_i + \beta_4 arrival_i^2$$

$$\alpha_j \sim Normal(\mu_\alpha, \sigma_\alpha)$$

2. JAGS code for the above model

```
model{

#likelihood

for(i in 1:nobs){

eld[i]~dnorm(mu.eld[i],tau.eld[i])

#model on mu
mu.eld[i]<-a.eld[id.eld[i]] + beta.arr*arr[i]

#model on sigma
tau.eld[i]<-1/sigma.eld[i]^2
log(sigma.eld[i])<-alpha.sigma + beta.arr.sig*arr[i] +
 beta.arr.sig2*arr[i]*arr[i]
}

#random intercept

for(j in 1:nobs.id){
a.eld[j]~dnorm(mu.id.eld, tau.id.eld)
```

```
}


#priors
mu.id.eld~dnorm(0, 0.0001)
alpha.sigma~dnorm(0, 0.0001)

beta.arr~dnorm(0,0.0001)
beta.arr.sig~dnorm(0,0.0001)
beta.arr.sig2~dnorm(0,0.001)

tau.id.eld<-1/sigma.id.eld^2

sigma.id.eld~dunif(0,20)

} #close model
```
